# Quantum Hall Effect across Graphene Grain Boundary

**DOI:** 10.3390/ma15010008

**Published:** 2021-12-21

**Authors:** Tuan Khanh Chau, Dongseok Suh, Haeyong Kang

**Affiliations:** 1 Department of Energy Science, Sungkyunkwan University, Suwon 16419, Korea; ctkhanh@skku.edu; 2 Department of Physics, Pusan National University, Busan 46241, Korea

**Keywords:** CVD graphene, grain boundary, quantum Hall effect, electrical transport

## Abstract

Charge carrier scattering at grain boundaries (GBs) in a chemical vapor deposition (CVD) graphene reduces the carrier mobility and degrades the performance of the graphene device, which is expected to affect the quantum Hall effect (QHE). This study investigated the influence of individual GBs on the QH state at different stitching angles of the GB in a monolayer CVD graphene. The measured voltage probes of the equipotential line in the QH state showed that the longitudinal resistance (*R*_xx_) was affected by the scattering of the GB only in the low carrier concentration region, and the standard QHE of a monolayer graphene was observed regardless of the stitching angle of the GB. In addition, a controlled device with an added metal bar placed in the middle of the Hall bar configuration was introduced. Despite the fact that the equipotential lines in the controlled device were broken by the additional metal bar, only the *R*_xx_ was affected by nonzero resistance, whereas the Hall resistance (*R*_xy_) revealed the well-quantized plateaus in the QH state. Thus, our study clarifies the effect of individual GBs on the QH states of graphenes.

## 1. Introduction

Graphene is an atomically thin monolayer that has attracted considerable attention in many fields due to its excellent mechanical [1,2] and electronic properties [3,4] that make it a promising material for next-generation nanoelectronics [3,5,6,7]. Significantly, the quantum Hall effect (QHE) in graphene [8,9,10], defined by a vanishing of longitudinal resistance (*R*_xx_) accompanied by the quantized plateau of Hall resistance (*R*_xy_), can be observed at up to room temperature [11]. Thus, the novel quantum transport behavior can be studied efficiently without the sophisticated growth of high-quality, thin epitaxial films. Moreover, the exact quantization value of QH resistance, *R*_K_/ν (*R*_K_ is the von Klitzing constant and ν is the filling factor with integer number), is utilized as the resistance standard in metrology [12,13,14]. The QH state in graphene can also be used to probe the electrochemical activity of oxygen vacancies in the graphene/functional oxide interface (Gr/STO) [15] and detect the hidden localized state at the interface of graphene on a hexagonal boron nitride (*h*-BN) substrate [16].

Chemical vapor deposition (CVD) graphene often forms grain boundaries (GBs) during CVD growth [17,18], consequently limiting the graphene quality. Theoretically, the electrical transport between single-crystal domains can be affected by scattering at the GB [19,20,21,22,23,24]. Previous reports have shown that GBs are the source of charge carrier scattering, thus degrading the performance of the related device [21,25,26,27]. Furthermore, a theoretical study of the QHE in polycrystalline graphene showed that the GBs destroy the QH state [28,29], and an experimental study of the QHE in polycrystalline CVD graphene showed strong backscattering of charge carriers in the QH regime [30]. The GB is a defect line consisting of a series of pentagonal, hexagonal, and heptagonal rings formed at the stitching region between two domains with different orientations [21,31,32]. The misorientation angle between two domains, referred to as the stitching angle, determines the structure and periodicity of the GB. However, there is a lack of studies on the QHE depending on the stitching angles of only a single GB.

In this study, we experimentally investigated the effect of the stitching angle of the GB on the QH state of graphene. It is remarkable that all the devices with a GB showed the standard QH states, which had the same sequence of filling factors as those of a single-crystal monolayer CVD graphene. This supports the fact that QH states are robust because of topological protection from scattering by the GB. In contrast, a controlled device with an additional electrode placed at the middle of the Hall bar structure was examined to show that the initial QH state is split into two QH states by the effect of the metal bar with different topologies. As a result, *R*_xx_ becomes nonzero because the equipotential line condition along the edges in the QH state is broken. However, each region separated by the metal bar maintained the typical quantized *R*_xy_. Our study provides an exemplary controlled experimental result for the effect of the GB on the QH state in a CVD graphene.

## 2. Materials and Methods

### 2.1. Graphene Growth

First, a 100 μm thick copper foil (from Nilaco, 99.96%, Tokyo, Japan) was annealed in forming gases (Ar, H_2_) at 1070 °C for 3 h and was polished using the chemical mechanical polishing method. Second, the polished copper foil was then heated up to 1070 °C with 200 sccm Ar gas and 100 sccm H_2_ gas in CVD chamber for annealing for 2 h to increase the crystallinity and remove residuals on the copper surface. The H_2_ was reduced to 40 sccm, and 15 sccm CH_4_ gas (0.1% diluted in Ar) was injected during graphene growth. Finally, CH_4_ gas was turned off after growth, and the chamber was cooled to room temperature.

### 2.2. Device Fabrication

The graphene device was fabricated on a heavily *p*-doped silicon substrate with a 300 nm thick SiO_2_ insulating layer via the following steps. First, a supporting polymer poly(methyl methacrylate) PMMA-C4 was spin-coated on the graphene/Cu substrate (3000 rpm/m). The PMMA-supported graphene film was then detached from the copper foil using the electrochemical bubbling transfer method and was rinsed in distilled water a few times to clean the residuals. Second, the PMMA/graphene was picked up by SiO_2_/Si substrate and dried on hot plate at 100 °C for 10 min. The PMMA was then removed by acetone and isopropyl alcohol. Finally, the graphene was subsequently etched by oxygen plasma (3 mTorr and 30 W) for D1, D2, D3, and D4 to fabricate the Hall bar structure and the graphene electrode. Metal electrode patterning was performed using electron-beam lithography, followed by Ti/Au (5/50 nm) evaporation under high-vacuum conditions (approximately 10^−6^ Torr). The graphene device was annealed in forming gas (H_2_, Ar) at 300 °C for 3 h to remove contamination on the graphene surface before performing electrical transport measurement.

### 2.3. Device Characterization

The Raman spectra were recorded with a 532 nm laser under ambient conditions (Renishaw Inc., New Mills, UK). The DC electrical characteristics of the graphene were analyzed under high vacuum (approximately 10^−6^ Torr) in a cryostat (Physical Property Measurement System (PPMS), Quantum Design Inc., San Diego, CA, USA) with a semiconductor parameter analyzer (4200SCS, Keithley Instruments Inc., Cleveland, OH, USA).

## 3. Results and Discussion

**Figure 1:**
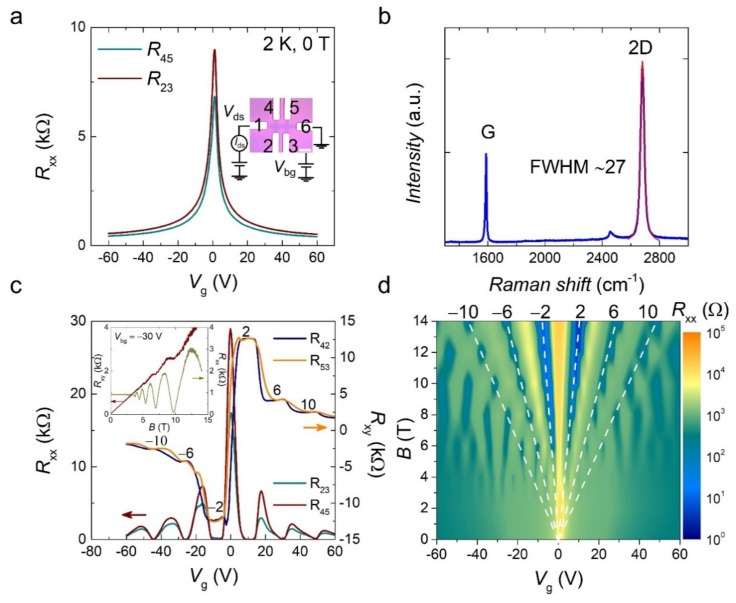
Single-crystal monolayer chemical vapor deposition (CVD) graphene device (D1) and its quantum Hall (QH) state. (**a**) Longitudinal resistance (*R*_xx_) at zero magnetic field and 2 K with an applied voltage of *V*_ds_ = 100 mV. The inset is the optical microscopy image of the device. The scale bar is 5 μm. (**b**) Raman spectra of monolayer graphene. The fitting of 2D peak is indicated by red line. (**c**) Gate voltage (*V*_g_) dependence of *R*_xx_ (*R*_42_, *R*_53_) and Hall resistance (*R*_xy_) (*R*_23_, *R*_45_) measured at 2 K and 14 T with an applied current of *I*_ds_ = 1 μA. The inset shows magnetic field dependence of *R*_xx_ and *R*_xy_ measured at *V*_bg_ = −30 V. (**d**) Landau fan diagram of *R*_xx_ as a function of *V*_g_ and magnetic field *B*; the white dashed lines are guides for the eye for Landau levels (LLs) with filling factors ν = ±2, ±6, and ±10.

All graphene flakes presented in this study were synthesized on a copper (Cu) foil using chemical vapor deposition (CVD) at 1070 °C with a mixture of hydrogen and methane gases. First, the graphene was transferred onto a silica (SiO_2_) substrate using the electrochemical bubbling transfer method [33]. Next, the metal electrodes were patterned by electron-beam lithography (EBL), followed by Ti/Au (5/50 nm) metal deposition. Finally, a Hall bar structure was defined by EBL, and reactive-ion etching was performed to remove redundant graphene. The final optical microscopy image of a single-crystal CVD graphene device (D1) is shown in the inset of Figure 1a with electrode numbers from 1–6. The typical ambipolar behavior of graphene was observed, and the charge neutrality point (CNP) was located around zero gate voltage, as shown in Figure 1a, indicating that the CVD graphene was free from an extrinsic doping effect. The field-effect mobility was also calculated, yielding *μ_FE_* = 12,000 cm^−2^ V^−1^ s^−1^. Raman spectroscopy was used to characterize the crystallinity of the graphene, as shown in Figure 1b. The monolayer graphene was confirmed by the intensity ratio of 2D/G peaks (~2) and the full width at half maximum (FWHM) of the 2D peak (~27) [34,35].

Figure 1c shows the gate voltage (*V*_g_) dependence of the longitudinal resistance (*R*_xx_) and the Hall resistance (*R*_xy_) at a magnetic field of 14 T and a temperature of 2 K. The *R*_xy_ in a monolayer graphene under a magnetic field is well known to be quantized according to $R_{xy}^{-1}=\pm g_{s}\left(n+1/2\right)e^{2}/h$ [9] where $g_{s}$ is the energy state degeneracy, *e* is the electron charge, *h* is Planck’s constant, *n* is a non-negative integer, and ± represents electrons and holes. This quantization can be translated into the quantized filling factor: $\nu =\pm g_{s}\left(n+1/2\right)$. The device exhibited a clear QHE with *R*_xx_ (*R*_23_, *R*_45_) moving toward zero and well-quantized plateaus in *R*_xy_ (*R*_42_, *R*_53_) with filling factors of ν = ±2, ±6, and ±10. We also measured the magnetic field dependence of *R*_xx_ and *R*_xy_ at a fixed gate voltage: *V*_g_ = −30 V, corresponding to the region of the hole-type carrier, as shown in the inset of Figure 1c. At a low magnetic field, *R*_xx_ was constant, and *R*_xy_ showed a linear dependence. However, as the magnetic field increased, *R*_xx_ showed an apparent oscillation, and *R*_xy_ showed quantized plateaus, reflecting the Landau levels (LLs) and QHE. It is convenient to visualize the QHE using a Landau fan diagram, where *R*_xx_ is plotted as a function of the magnetic field *B* and *V*_g_ (Figure 1d). As a result, the electrical transport of the device in the presence of a magnetic field follows the standard QHE of a monolayer graphene device. Therefore, the observation of a clear QHE indicates that the CVD graphene is of high enough quality to study the effect of GBs on the QHE, excluding other issues.

**Figure 2:**
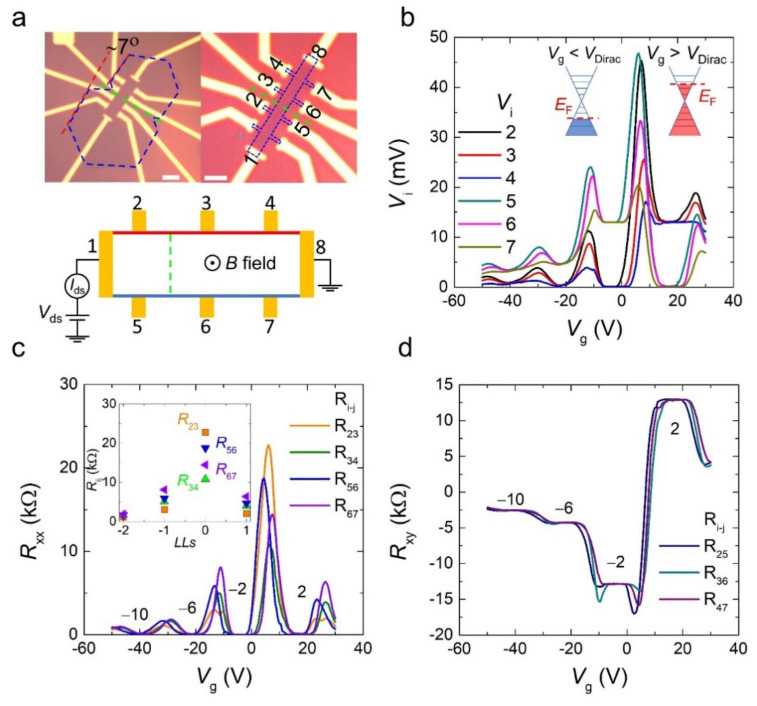
The quantum Hall (QH) state of graphene device (D2) with grain boundary (GB) at 2 K and 14 T with an applied current of *I*_ds_ = 1 μA. (**a**) Optical microscopy image of the device (top) and edge state potential of the device. The graphene is denoted by the blue dashed line. The green dashed lines indicate the GB position. The stitched angle is approximately 7° and indicated by red dashed lines. The scale bar is 3 μm. (**b**) Gate voltage dependence (*V*_g_) of potentials measured at each voltage probe. (**c**,**d**) *V*_g_ dependence of *R*_xx_ and *R*_xy_, respectively.

The GB was obtained at the interface when two hexagonal graphene domains were merged without any additional treatment or fabrication process. In this situation, the location of the GB can be easily identified by simple optical microscopy [36], as indicated in Figure 2a. The blue, green, and red dashed lines correspond to two hexagonal graphenes, the location of the GB and the stitching angle, respectively. We placed multiple electrodes along the device (D2) to simultaneously study the QH states in the areas containing and not containing GB with a stitching angle of ~7°. Figure 2b shows the gate dependence of voltage (*V*_i_) at each electrode probe measured when the current (*I*_ds_) was injected through electrodes 1–8 (schematic in Figure 1a) at 14 T and 2 K. From the set of voltage values, we extracted the longitudinal resistance: *R*_xx_ = (*V*_i_ − *V*_j_)/*I*_ds_, where i and j are the probes at the same edge, such as *R*_23_, and the Hall resistance: *R*_xy_ = (*V*_i_ − *V*_k_)/*I*_ds_, where i and k are the probes facing each other, such as *R*_25_, as a function of the gate voltage sweep, as shown in Figure 2c,d. The behaviors of *R*_xx_ and *R*_xy_ are very similar to those shown in Figure 1c. Here, we extracted the peak values of *R*_xx_, which occurred whenever the filling factor changed, as depicted in the inset of Figure 2c. Noteworthy, all *R*_xx_ pairs had the same channel length and width. At the zero Landau level, the resistance peaks of the electrode pairs across the GB (*R*_23_ and *R*_56_) were higher than those that did not contain the GB (*R*_34_ and *R*_67_) because of scattering at the GB, which is consistent with previous reports [36,37]. However, the resistance peaks at higher Landau levels did not follow this tendency. It seems that scattering at the GB only occurs at a low carrier concentration which can be understood as the change of energy level far away from the defect states generated from the chemical adsorption, such as O and OH radicals at the GB [38]. However, all pairs of *R*_xy_ show well-quantized Hall plateaus accompanied by *R*_xx_ = 0 Ω at filling factors ν = ±2, ±6, and ±10, indicating the standard QHE of a monolayer graphene.

To clarify the influence of the GB on the QHE, we focused on the potential profiles of the sample. In general, because backscattering is suppressed in the QH state, the equipotential along the edge is generated, which is distinguished from the metallic state. When the direction of the applied current and magnetic field is fixed, both holes and electrons accumulate at the same edge of the device. Therefore, the sign of the potential changes depends on the carrier type. Interestingly, in Figure 2b, the measured voltage values along the edge are the same; for example, *V*_2_ *= V*_3_ = *V*_4_ when *V*_g_ > *V*_Dirac_ and *V*_5_ = *V*_6_ = *V*_7_ when *V*_g_ > *V*_Dirac_, even though the GB existed between electrodes 2(5) and 3(6). The voltage probe showed that the potential line in the device was similar to the standard QH state, just as we drew the equipotential lines in blue and red, as shown in the schematic in Figure 2a. Therefore, our results reveal that the GB does not affect the edge states of topologically protected QH regimes.

**Figure 3:**
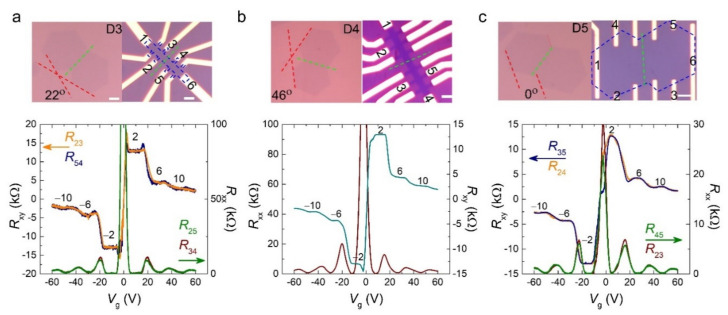
Quantum Hall (QH) state of grain boundary (GB) graphene device at different stitching angles. (**a**–**c**) Optical microscopy image of the device (top) and gate voltage (*V*_g_) dependence of *R*_xx_ and *R*_xy_ (bottom) of the devices with stitched angles 22°, 46°, and 0°, respectively. The graphene channel, GB, and stitching angle are denoted by the blue, green, and red dashed lines, respectively. The data were measured at 14 T and 2 K with an applied current of *I*_ds_ = 1 μA. The scale bars are 4 μm.

As mentioned previously, the details of defect lines in the GB can be different from the stitching angle between two graphene domains. Therefore, it can be another parameter to consider in the scattering mechanism and its effect on the QH state. We fabricated three more devices with different stitching angles, as shown in Figure 3. Graphenes in devices D3 and D4 (Figure 3a,b) were etched to make the Hall bar structure with stitching angles of 22° and 46°, respectively. However, device D5 had a zero stitching angle of two hexagonal graphene domains, and it was completed without an etching process. The electrical transport of all the devices at 14 T and 2 K showed the standard QHE of a monolayer graphene. Moreover, *R*_xy_ measured with two electrodes passing through the GB, such as *R*_24_ in Figure 3a, also supports other *R*_xy_ results, which indicate the equipotential along the edge, regardless of the stitching angle. Therefore, this confirms that the topologically protected QH state is robust and rarely affected by GB scattering.

**Figure 4:**
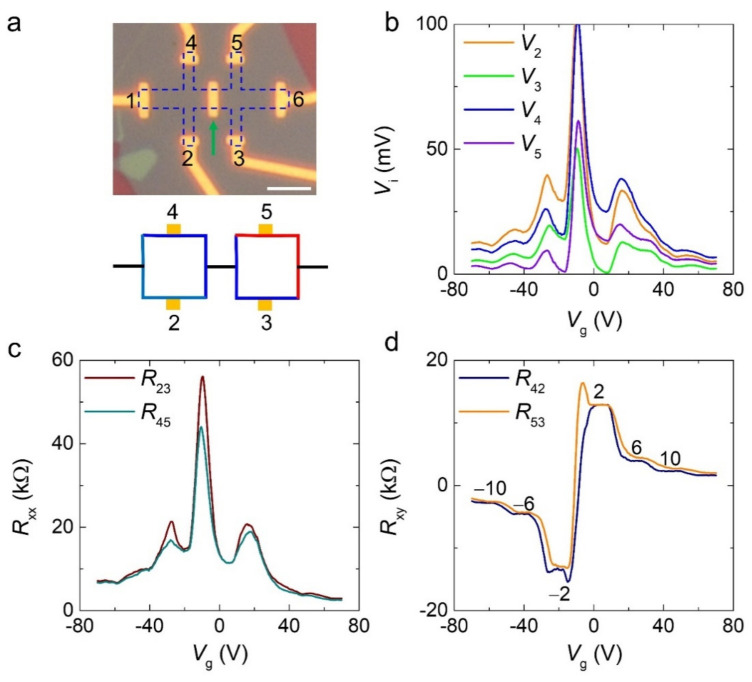
Quantum Hall (QH) state of the controlled device with an additional electrode placed at the middle of the Hall bar structure device. (**a**) Optical microscopy image of the device (top) and chemical potential line of the device in the presence of magnetic field (14 T) at low temperature (2 K). A blue dashed line denotes the graphene channel, and a green arrow indicates the additional metal bar. The scale bar is 5 μm. (**b**) Gate voltage (*V*_g_) dependence of each voltage probe. (**c**,**d**) *V*_g_ dependence of *R*_xx_ and *R*_xy_, respectively. The data were measured at 14 T and 2 K with an applied current *I*_ds_ = 1 μA.

To contrast the effect of the edge states on the QHE, we broke the equipotential line along the edges by placing an additional metal electrode at the center of the Hall bar structure of a single-crystal monolayer CVD graphene device on a hexagonal boron nitride (*h*-BN) substrate. The optical microscopy image of the device is shown in Figure 4a (top), with the metal bar indicated by the green arrow. The added electrode can be considered as an additional electrical probe in a normal metallic state and does not change the measurement configuration. However, the potential in the QH state abruptly decreased through this electrode. Thus, one QH state (without an additional electrode) was divided into two equivalent QH states (with an additional electrode). Figure 4b shows the voltage (*V*_i_) measured at each electrode probe as a function of the gate sweep at 2 K and 14 T. Unlike the equipotential line of a typical QH state in the graphene device, as shown in Figure 2b, *V*_5_ was not equal to *V*_4_ (*V*_g_ > *V*_Dirac_) but equal to *V*_2_ at the first LL (ν = ±2), which made *R*_45_ nonzero in the QH state, as shown in Figure 4c. However, *V*_5_ was not equal to *V*_2_ at higher LLs, indicating that the equipotential lines were broken at high carrier concentrations. The *R*_xy_ shows the standard sequence of the filling factors ν = ±2, ±6, and ±10, as shown in Figure 4d. The equipotential line of the device based on the measurement data in Figure 4b is depicted as a schematic in Figure 4a (bottom).

## 4. Conclusions

We studied the effect of a single GB in four different devices (D2, D3, D4, and D5) at various stitching angles (0°, 7°, 22°, and 46°) on the electrical transport in a CVD graphene, especially in the QH regime. In the device D2 with the stitching angle of 7°, the scattering at the GB in the longitudinal resistance (*R*_xx_) appears to be dominant only in the low carrier concentration region as we compared the peaks resistance at the Landau levels. Furthermore, it does not disrupt the dissipationless edge states, showing the standard QHE in the monolayer graphene, as evidenced by the ideal equipotential lines detected by the voltage measurement of each probe and the well-quantized Hall plateaus at filling factor ν = ±2, ±6, ±10, regardless of the stitching angles. We also compared the results of the device that artificially broke the QH states. As a result, the distortion of equipotential lines caused by the broken QH states only affects *R*_xx_ with nonzero resistance, while *R*_xy_ showed the well-quantized Hall plateaus_._ Therefore, we postulate that topologically protected QH states are robust, even when an individual GB is present.

## Data Availability

The data presented in this study are available on request from the corresponding author.
